# Beyond inhibition: lateral modulation of plasticity of feedforward synapses in a spiking model of V1

**DOI:** 10.1186/1471-2202-14-S1-P187

**Published:** 2013-07-08

**Authors:** Csaba Petre, Micah Richert, Botond Szatmary, Eugene Izhikevich

**Affiliations:** 1 Brain Corporation, San Diego, CA, 92121, USA

## 

Lateral inhibition is typically used to repel neural receptive fields. Here we introduce an additional learning mechanism that modifies the plasticity for feedforward synapses based on lateral interactions. We show a model, based on evidence from biological recordings[1], in which a heterosynaptic learning rule in conjunction with lateral inhibition introduces competition among neurons for input features.

We demonstrate an STDP learning rule for feedforward connections to a spiking neuron where plasticity is modulated by activity of neighboring neurons. We apply the learning rule to a spiking model of primary visual cortex. In our model, input to V1 cortical neurons comes from the magnocellular pathway of a simulated spiking retina responding to saccades over a natural image. A group of neurons in V1 respond to a particular input feature and convey information that they have spiked to neighboring neurons by way of specialized lateral connections. The connections convey a direct signal of recent spiking activity. Alternatively, lateral inhibitory synapses and the level of inhibition to a neuron can also be used as this signal. If this recent neighbor activity signal is high, feedforward plasticity to the neuron becomes a simple flat depression as opposed to regular exponential STDP, as shown in Figure 1A. Thus, late spiking neurons are prevented from developing receptive fields similar to those of earlier spiking neurons for a given input feature. This rule in conjunction with lateral inhibition and slow weight updrift ensures competition between neurons for features over a long timescale.

**Figure 1:**
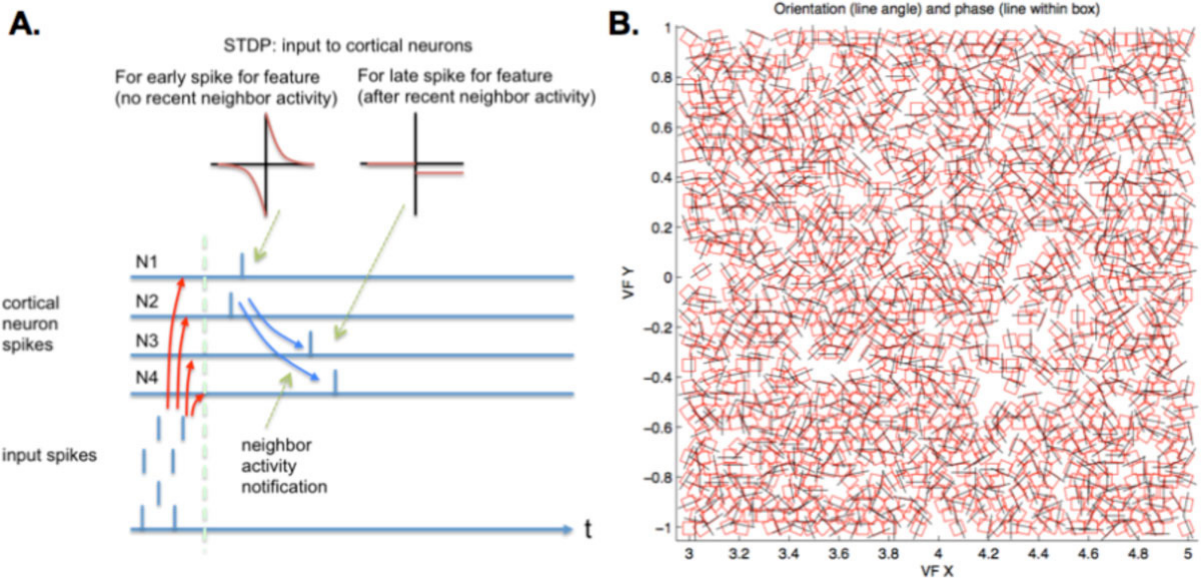
**A. Detail of the heterosynaptic rule**. Neurons N1 and N2 fire early for the input, and their feedforward synapses undergo normal STDP. They send an activity signal to N3 and N4, whose feedforward synaptic weights are then depressed to prevent learning of the same feature. **B**. Map of measured emergent orientation and phase responses of neurons in a 2 × 2 degree section of V1 layer 4 of our spiking V1 model. We observe a wide range of oriented feature selectivity and good spatial coverage of the feature space.

Our V1 model is built on a spatially distributed grid of single-compartment spiking neurons with parameters configured to model regular spiking cortical pyramidal cells, as detailed in [2]. The model has plastic excitatory feedforward connections and local lateral inhibition between spatially proximal neurons. In our model, we observe a better overall coverage of orientation selectivity of V1 Layer 4 neurons with our heterosynaptic rule than without it. We find a larger range of orientations and less redundancy of receptive field features between neighboring neurons, as shown in Figure 1B. Such a rule could be used in a generic spiking cortical architecture to enforce independence of neural receptive fields.

## References

[B1] Chistiakova M Volgushev C Heterosynaptic plasticity in the neocortex Exp Brain Res 2009 199 377 390 10.1007/s00221-009-1859-5 19499213 PMC2781103

[B2] Izhikevich EM Simple model of spiking neurons IEEE Transactions on Neural Networks 2003 199 377 390 10.1109/TNN.2003.820440 18244602

